# Regulatory T Cell Heterogeneity in the Thymus: Impact on Their Functional Activities

**DOI:** 10.3389/fimmu.2021.643153

**Published:** 2021-02-11

**Authors:** Jérémy C. Santamaria, Alexia Borelli, Magali Irla

**Affiliations:** Aix-Marseille University, CNRS, INSERM, CIML, Centre d'Immunologie de Marseille-Luminy, Marseille, France

**Keywords:** immune tolerance, regulatory T cells, thymus, thymic recirculation, regulatory T cell heterogeneity, autoimmune disorders

## Abstract

Foxp3^+^ regulatory T cells (Treg) maintain the integrity of the organism by preventing excessive immune responses. These cells protect against autoimmune diseases but are also important regulators of other immune responses including inflammation, allergy, infection, and tumors. Furthermore, they exert non-immune functions such as tissue repair and regeneration. In the periphery, Foxp3^+^ Treg have emerged as a highly heterogeneous cell population with distinct molecular and functional properties. Foxp3^+^ Treg mainly develop within the thymus where they receive instructive signals for their differentiation. Recent studies have revealed that thymic Treg are also heterogeneous with two distinct precursors that give rise to mature Foxp3^+^ Treg exhibiting non-overlapping regulatory activities characterized by a differential ability to control different types of autoimmune reactions. Furthermore, the thymic Treg cell pool is not only composed of newly developing Treg, but also contain a large fraction of recirculating peripheral cells. Here, we review the two pathways of thymic Treg cell differentiation and their potential impact on Treg activity in the periphery. We also summarize our current knowledge on recirculating peripheral Treg in the thymus.

## Introduction

CD4^+^Foxp3^+^ regulatory T cells (Treg) crucially maintain immune tolerance. These cells are endowed with the unique ability to protect from life-threatening autoimmune reactions and dampen inflammatory responses. The absence of Foxp3^+^ Treg in IPEX (Immune dysregulation, Polyendocrinopathy, Enteropathy, X-linked syndrome) patients, and scurfy mice results in a severe lymphoproliferation and multi-organ autoimmunity (1–4). Treg cells are also involved in numerous physio-pathological processes such as foeto-maternal tolerance, allergy, anti-tumoral immunity, tissue homeostasis, and repair (5, 6). This large array of functions implies a division of labor by several Treg subsets. This heterogeneity is illustrated by the central Treg (cTreg)/effector Treg (eTreg) dichotomy (7). cTreg are found in lymphoid organs and depend on IL-2, whereas eTreg populate non-lymphoid tissues and depend on TCR signals for their proliferation. Similarly to conventional CD4^+^ T helper (Th) cells, the cytokine microenvironment induces the polarization of Treg into Th1-, Th2-, Th17-, and follicular Treg (Tfr)-like cells, showing distinct chemokine receptor expression patterns (8). This polarization allows Treg to specifically antagonize their conventional CD4^+^ Th counterparts.

The majority of Foxp3^+^ Treg develop in the thymus (tTreg) although conventional CD4^+^ T cells can also convert in the periphery into Foxp3^+^ Treg under certain conditions (pTreg) (9). Several precursors including CD25^+^Foxp3^−^ cells, recent thymic emigrants (RTE) and FR4^hi^CD73^hi^ cells have been proposed to differentiate into pTreg, which have both overlapping and unique TCR repertoire and functions compared to tTreg (9–17). Thymic development of Treg cells was initially suggested by seminal work during the 1960's describing generalized autoimmune manifestations in neonatal thymectomies, before day 3 but not later (18, 19). Further works demonstrated that suppressive T cells exerting a dominant tolerance develop during this neonatal period in the thymus and were called “regulatory T cells” (20–24). Thymic Treg cells represent only ~2–3% of developing CD4^+^ single positive (SP) thymocytes. Their development is driven by strong TCR stimulation coupled to the CD28 co-stimulatory signal. A third signal mediated by γ-chain cytokines promotes their survival and progression into a mature CD25^+^Foxp3^+^ Treg stage (25).

Recent advances have revealed that thymic Treg constitute a more diverse and dynamic population than previously thought. Emerging evidences indicate that the phenotypically and functional diversity of Treg could be imprinted during their ontogeny in the thymus. In this review, we discuss our current knowledge on the two identified precursors leading to the development of mature Treg, showing distinct regulatory activities. We also highlight a new population of mature Treg recirculating from the periphery into the thymus.

## Distinct Thymic Foxp3^+^ Treg Subsets Generated by Two Developmental Pathways

**Figure 1:**
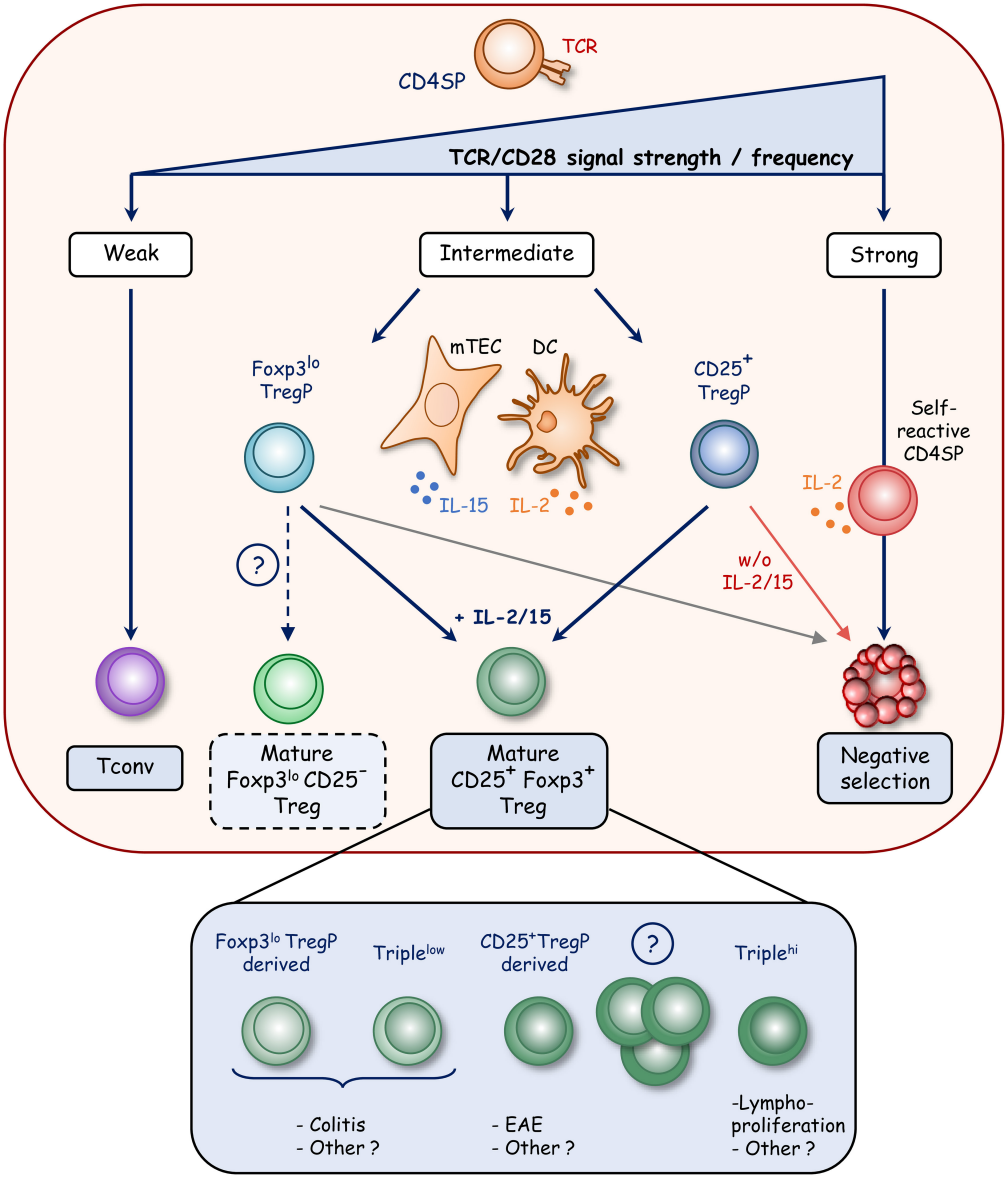
Model of Foxp3^+^ Treg development in the thymus. During thymic development, strong TCR signals lead to clonal deletion of highly self-reactive CD4^+^ SP thymocytes whereas weak signals result in the generation of conventional T cells (Tconv). In contrast, intermediate TCR signals result in the diversion of CD4^+^ SP thymocytes into the Treg cell lineage with the development of two distinct precursors. CD25^+^ Treg precursors (CD25^+^ TregP) develop upon strong TCR signals that induce CD25 expression whereas Foxp3^lo^ Treg precursors (Foxp3^lo^ TregP) develop upon weaker TCR signals, which drive the upregulation of Foxp3. Further development of these two precursors requires survival signals mediated by γc cytokine signaling. IL-2 produced by DC and self-reactive CD4^+^ SP thymocytes and IL-15 produced by mTEC rescue Treg precursors from cell death and lead to mature CD25^+^Foxp3^+^ Treg development. Interestingly, mature CD25^+^Foxp3^+^ Treg appear to have distinct functional properties according to the precursors from which they are derived. CD25^−^Foxp3^lo^ cells also likely contain a subset of mature Treg that egress to the periphery (dotted line). Mature Treg also encompass Triple^hi^ (GITR^hi^PD1^hi^CD25^hi^) and Triple^lo^ (GITR^lo^PD1^lo^CD25^lo^) Treg subsets, which show distinct functional activities.

T-cell fate decision toward clonal deletion (also called negative selection) or the diversion into the Treg or T conventional cell lineage is dictated by TCR affinity and/or avidity for self-peptides presented by thymic antigen presenting cells [APC (26)]. The relative contributions of the different APC subsets such as medullary thymic epithelial cells (mTEC), cortical TEC (cTEC), B cells, conventional, and plasmacytoid dendritic cells (DC) in T-cell fate decision remain poorly understood although bone marrow-derived APC and mTEC seem to play non-overlapping roles in shaping the TCR repertoire (27). The introduction of a cognate antigen in TCR transgenic mice leads to both clonal T-cell deletion and Treg induction, indicating that the diversion into the Treg cell lineage is also induced by the recognition of agonist self-peptide (28). Treg thus show a highly self-reactive TCR repertoire, distinct from conventional T cells, which contributes to their functional diversity (29–32). The prevailing paradigm of Treg development is based on the integration of signals mediated by TCR/CD28 stimulation and cytokine signaling. It relies on a “two-step model” in which strong agonist TCR signals in CD25^−^Foxp3^−^ CD4^+^ SP thymocytes results in the induction of CD25 (also known as IL-2Rα) and thus in the development of CD25^+^Foxp3^−^ Treg precursors (CD25^+^ TregP) (step one) (Figure 1). However, the TCR signal strength required to select Treg was described to be lower than that leading to clonal deletion of CD4^+^ SP thymocytes (30, 33). This could be due to a lower intrinsic TCR affinity or to the limited expression of the cognate antigen by APC, thus reducing multiple antigen encounters (34, 35). The CD28-CD80/86 costimulatory axis is also essential for Treg development from their precursor stage (36–38). This is well illustrated in *Cd28*^−/−^ and *Cd80*^−/−^ × *Cd86*^−/−^ mice showing impaired generation of CD25^+^ TregP and a severe defect in mature Treg. Furthermore, the TCR signaling pathway in association with the CD28 co-stimulation signal drives the upregulation of tumor-necrosis factor receptor superfamily (TNFRSF) members such as GITR, OX40, and TNFR2 in CD25^+^ TregP (39). The CD28 co-stimulation in developing thymocytes has also been reported to induce Foxp3 expression (40).

The second step of Treg development that drives the maturation of CD25^+^ TregP into mature CD25^+^Foxp3^+^ Treg depends on γ-chain cytokines, in particular IL-2 and/or IL-15 signaling (Figure 1) (41–43). Mice deficient for the IL-2rγ chain, which is shared by IL-2,-4,-7,-9,-15, and -21 receptors, lack this cell type (42, 44). However, mice deficient for IL-2 or IL-2rα display only half of Foxp3^+^ Treg, indicating that IL-2 plays a major role in Treg development (44). Furthermore, mice lacking the IL-2rβ chain, shared by IL-2 and IL-15 receptors, were also found to almost lack mature Treg (42, 44, 45). Accordingly, the generation of IL-2 × IL-15 double-deficient mice has revealed a similar Treg defect as that observed in *Il2r*β^−/−^ mice, indicating that IL-15 is also implicated in Treg cell development (42, 46, 47). These two cytokines promote survival and thus rescue Treg from the pro-apoptotic effects of Foxp3 (48). In addition, the stimulation of GITR, OX40, or TNFR2 participates in this second step by enhancing the conversion of CD25^+^ TregP into mature Treg through an increase responsiveness to IL-2 (39).

Recent advances have shed new light on the cellular source of IL-2 and IL-15 cytokines. DC have been initially proposed to constitute a privileged developmental niche for Treg cell generation by providing both cognate self-antigens and IL-2 (49). However, this concept has been recently challenged. Although IL-2 can be produced by thymic DC, the selective ablation of the *Il2* gene in DC and T cells in the absence of IL-15 revealed that only IL-2 provided by T cells severely impairs Treg development (50). Accordingly, IL-2 production was more precisely assigned to self-reactive CD4^+^ thymocytes, including Treg precursors (51). These observations suggest that the size of the Treg cell population is regulated by self-reactive thymocytes through IL-2 production. In contrast to IL-2, IL-15 is provided by stromal cells, mainly TEC including cTEC and mTEC expressing both low and high levels of MHC class II molecules (46, 52, 53).

A second developmental pathway has recently emerged based on the identification of another Treg precursor subset expressing low level of Foxp3 and lacking CD25 cell surface expression (Foxp3^lo^ TregP) (46, 54). Similarly to CD25^+^ TregP, a substantial proportion of Foxp3^lo^ TregP differentiates into mature Treg upon intrathymic transfer. These observations indicate that both CD25^+^ and Foxp3^lo^ TregP contribute to the generation of the mature Treg pool. Foxp3^lo^ TregP seems to depend on IL-15 for their survival, and then on IL-2 to fully differentiate into mature Treg cells whereas CD25^+^ TregP likely depend on IL-2 for their development. Treg cells are thus generated *via* two distinct developmental programs involving CD25^+^ and Foxp3^lo^ TregP (54) (Figure 1). CD25^+^ TregP show a higher affinity for self-antigens than Foxp3^lo^ TregP. Accordingly, these two precursors exhibit a distinct TCR repertoire and thus make unique contribution to the mature Treg TCR repertoire. Furthermore, they have distinct transcriptomes, regulated by different signaling pathways and enhancers. The NFκB signaling pathway and the Foxp3 regulatory element *Cns3* were found to be required for the development of Foxp3^lo^ TregP but dispensable for CD25^+^ TregP. Interestingly, although these two precursors can give rise to mature Treg, they have distinct functional capacities. Treg derived from CD25^+^ TregP protect from experimental auto-immune encephalomyelitis, whereas those derived from Foxp3^lo^ TregP protect from T-cell induced colitis. These observations open new perspectives to treat autoimmune disorders by Treg-cell based therapy, which is currently extremely promising in clinical trials (55). Moreover, single-cell transcriptomic analysis suggests the presence of CD25^+^ and Foxp3^lo^ TregP in the human thymus (56), indicating conserved developmental pathways, although further investigations are required.

The discovery of these two Treg cell precursors raises the question about the existence of a common or a distinct pre-precursor. It has been recently proposed that CD4^+^GITR^+^CD122^+^CD25^−^Foxp3^−^ cells represent a Treg pre-precursor population, which remains to be clearly established (57, 58). Furthermore, CD25^−^Foxp3^lo^ cells seem to be at a more mature stage than CD25^+^Foxp3^−^ cells based on their CD24^lo^Qa2^hi^ phenotype or their “thymic age” measured by their green fluorescent protein (GFP) expression level using transgenic mice expressing GFP under the recombination-activating gene 2 promoter (*Rag2-*GFP model) (59). These observations could be due to a later emergence of these cells during T-cell development or by the presence of mature developing Treg among this population as suggested by other experiments (60). In accordance with this latter hypothesis, a substantial fraction of RTE and functional peripheral Treg are CD25^−^Foxp3^+^ cells (7, 46, 61). Thus, further investigations are required to better define the cellular composition of the CD25^−^Foxp3^lo^ compartment.

Two mature Treg cell subsets were also identified based on the expression level of CD25, GITR, and PD-1 and were defined as GITR^lo^PD-1^lo^CD25^lo^ (Triple^lo^) and GITR^hi^PD-1^hi^CD25^hi^ (Triple^hi^) cells (61). These cells develop within the thymus and show a distinct TCR repertoire with different affinities for self-antigens with Triple^hi^ Treg being more self-reactive than Triple^lo^ Treg. Interestingly, Triple^hi^ Treg control the lympho-proliferation in peripheral lymph nodes, suggesting an ability to protect against auto-immune disorders, whereas Triple^lo^ Treg limit the development of colitis by promoting the conversion of conventional CD4^+^ T cells into pTreg. Nevertheless, their respective relationship with CD25^+^ and Foxp3^lo^ TregP remains to be established. Therefore, these recent advances on Treg development have revealed a developmentally regulated heterogeneity in the thymic Treg population (Figure 1). These findings thus indicate that the thymus generates multiple Treg subsets with non-overlapping regulatory activities.

## Heterogeneity and Function of Recirculating Thymic Treg Cells

**Figure 2:**
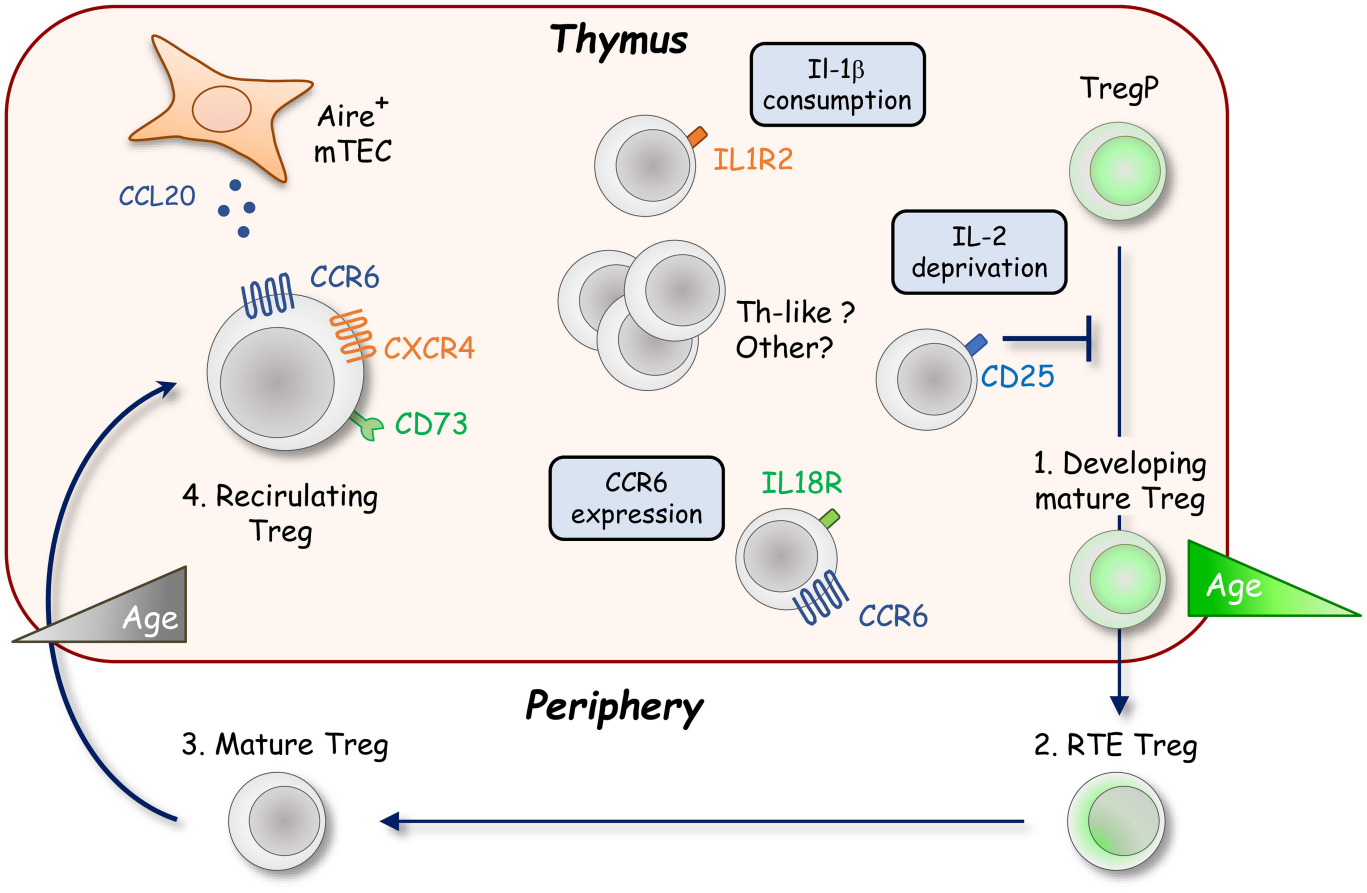
Recirculation of peripheral Foxp3^+^ Treg into the thymus. The thymic Foxp3^+^ Treg population is not only composed of developing *Rag2-*GFP^+^ Treg (green cell) but also contains a large fraction of recirculating mature *Rag2-*GFP^−^ Treg from the periphery showing an activated phenotype (gray cells). The recirculation of peripheral Treg likely depends on CCR6 and CXCR4 chemokine receptors. These cells mainly localize into the thymic medulla in response to the production of the CCR6 ligand, CCL20, by Aire^+^ mTEC. Once in the thymus, recirculating CCR6^+^CD73^+^ Treg inhibit the development of *de novo* CD25^+^Foxp3^+^ Treg in an age dependent manner by IL-2 deprivation. Distinct subsets of recirculating Treg were recently described such as IL-1R2^+^ Treg that maintain Treg development by quenching IL-1β under inflammatory conditions. An IL-18R^+^ subset, endowed with a higher ability to recirculate into the thymus than their IL-18R^−^ Treg counterparts, was also described. In this subset, IL-18 signaling likely promotes the thymic entry of peripheral Treg by upregulating CCR6 expression.

Although thymic Treg were thought to represent a homogenous population of developing cells for a long time, several recent studies have described the presence of mature cells derived from the periphery or in retention upon their development in the thymus. The homing of mature peripheral Treg into the thymus was initially reported in the context of parabiosis experiments and lymphopenic mice (62, 63). The generation of *Rag2-*GFP mice was particularly useful to distinguish developing *Rag2-*GFP^+^ Treg and older *Rag2-*GFP^−^ Treg (59). After positive selection, the expression of the *Rag2* gene stops and GFP undergoes exponential decay with a half-life of ~56 h (64). Using this model, a consequent fraction of thymic Treg were found to be *Rag2-*GFP^−^ cells (65). It was later demonstrated that these cells contain peripheral Treg migrating back to their organ of origin (Figure 2) (66, 67). Some *Rag2-*GFP^−^ Treg can also correspond to resident cells that have never left the thymus, making more complex the heterogeneity of this mature Treg population (68). Interestingly, mature *Rag2-*GFP^−^ Treg represent around half of the thymic Treg pool in 8 week-old B6 mice, and almost their totality in 6 month-old B6 mice, unraveling an age-dependent accumulation of these cells within the thymus (66, 68). These cells display an activated and effector phenotype, which is required for their migration into the thymus (69). Their trafficking is likely directed by CXCR4/CXCL12 and CCR6/CCL20 chemokine axes (66, 70). CCL20 is mainly produced by mTEC under the control of the transcription factor Aire (Autoimmune regulator), which is crucial for the establishment of T-cell tolerance (71). The CCR6/CCL20 axis could thus be responsible of the almost exclusive medullary localization of recirculating Treg (69, 70). It was recently described that the IL-18 stimulation of peripheral IL-18R-expressing Treg allows the expression of CCR6 that favors their recirculation into the thymus (72). In addition to CCR6, CD73 was found to discriminate mature *Rag2-*GFP^−^ Treg from newly produced *Rag2-*GFP^+^ Treg (54, 69). Recirculating mature Treg can thus be identified as CCR6^+^CD73^+^ in the absence of *Rag2-*GFP transgenic reporter mice (Figure 2).

However, is this recirculation an epiphenomenon or do these cells have a function in the thymus? The medullary localization, the effector phenotype and the accumulation of mature *Rag2-*GFP^−^ Treg during aging that occurs concomitantly with the reduced development of *Rag2-*GFP^+^ Treg suggests a suppressive function of these cells. In line with this hypothesis, the addition of mature Treg either to fetal thymic organ cultures (FTOC) or adult thymic tissue slices inhibits *de novo* Treg production by consuming locally available IL-2 (49, 66). Accordingly, an increase in Treg development is observed if the amount of recirculating Treg is naturally (different mouse strains) or artificially (peripheral CD4^+^ T-cell depletion) diminished (69). Thus, the thymic Treg niche seems to be stable with age, but dynamic in its composition and regulated by the amount of mature Treg that repopulate the thymus. This feedback loop regulation of Treg production by recirculating cells has been recently challenged by observations made in *Aire*^−/−^, *Ccr6*^−/−^, and *Il18r1*^−/−^ mice, which all show a decrease in Treg recirculation with no obvious impact on *de novo* generated Treg cells (70, 72). These observations could question the dynamic balance between developing and recirculating Treg. However, Treg recirculation is only partially abrogated in these mice and it cannot be excluded that the remaining recirculating mature Treg cells are able to inhibit Treg production. Furthermore, since numbers of Aire^+^ mTEC were reported to influence antigen-mediated Treg selection, the expected stronger Treg development in *Aire*^−/−^ mice could be hidden by impaired basal Treg induction, which was described in neonatal mice lacking recirculating Treg (73). Conversely, mice deficient for OPG (osteoprotegerin; encoded by *Tnfrsf11b*), the soluble decoy receptor for RANKL (receptor activator of nuclear factor-κB ligand; encoded by *Tnfsf11*), which controls the development of Aire^+^ mTEC, show an increase Treg recirculation with no defect in *de novo* Treg production (74). Nevertheless, the strong inhibition by recirculating Treg in these mice could be counterbalanced by an increased development of *de novo* Treg mediated by exacerbated Aire^+^ mTEC numbers (27, 75–77). Further studies are definitely required to clarify the impact of peripheral Treg on *de novo* Treg production. The generation of transgenic mice either fully lacking recirculating Treg, or with adjustable amount of recirculating Treg into the thymus, is expected to clarify this issue.

Since Treg promote the homeostasis of several other tissues such as skeletal muscle, visceral adipose tissue, lung, central nervous system, and skin (5, 6), recirculating Treg could also participate in thymus homeostasis. In particular, the crosstalk with CD4^+^ SP thymocytes controls the development of Aire^+^ mTEC that are responsible for peripheral Treg re-entry into the thymus (78, 79). Since recirculating Treg express RANKL and that the development of Aire^+^ mTEC is mediated by the RANK-RANKL signaling pathway (75, 79–81), it would be of special interest to define whether recirculating Treg are in turn implicated in the regulation of Aire^+^ mTEC cellularity or other stromal cells throughout lifespan. In line with this hypothesis, excessive RANK signaling driven by activated CD4^+^ T cells has been reported to enhance Aire^+^ mTEC development and *in fine* promote the exhaustion of epithelial progenitors (82). Further investigations are required to evaluate the function of mature recirculating Treg in the thymus. One important step before is undoubtedly to better characterize the cell subsets that reside within the recirculating mature Treg compartment. Similarly to developing Treg, a heterogeneity is emerging among recirculating mature Treg. Two subsets, IL-18R^−^, and IL-18R^+^ cells, the latter showing molecular similarities with tissue-resident effector Treg, have been described (72). Based on the differential expression of the decoy receptor for the inflammatory cytokine IL-1, IL-1R2^−^, and IL-1R2^+^ recirculating Treg have been also reported with IL-1R2^+^ Treg protecting Treg development under inflammatory conditions by quenching IL-1 (83). Furthermore, the Triple^hi^ and Triple^lo^ subsets described in the developing Treg compartment were also observed among recirculating Treg (61). However, the relationship between IL-18R^−^, IL-18R^+^, IL-1R2^−^, IL-1R2^+^, Triple^hi^, and Triple^lo^ subsets in recirculating Treg remain to be determined. Interestingly, high-throughput RNA sequencing on the bulk of *Rag2-*GFP^−^ Treg revealed a high expression for many genes specific of Th1-, Th2-, Th17-, Tfr-like Treg and markers found in adipose- and muscle-tissue Treg (Figure 2) (66). These observations suggest a greater heterogeneity in the *Rag2-*GFP^−^ Treg compartment than currently described. Single cell transcriptomic analysis of recirculating Treg is expected to shed new light on the composition of distinct effector cells that resides in this compartment. Interestingly, peripheral Treg also likely migrate back to the thymus in humans, indicative of a conserved mechanism. These cells, identified as CD31^−^, a marker expressed by developing thymocytes and RTE and downregulated during peripheral maturation, display an activated and differentiated phenotype (66). Further investigations are now required to better understand human mature Treg heterogeneity and functionality.

## Concluding Remarks

Recent advances have highlighted an unsuspected heterogeneity in the thymic Treg cell population, including in developing and recirculating Treg. Given that at least two precursors give rise to several subsets of mature Foxp3^+^ Treg, with distinct abilities to suppress autoimmunity, it would be not surprising that a functional imprinting is acquired from Treg cell development in the thymus. Beyond the TCR repertoire, this imprinting could be driven in specific niches providing different molecular and cellular cues such as cytokines, TNFRSF signals, and APC subsets. A heterogeneity in the recirculating Treg compartment is also currently emerging, opening new interesting questions on their tissue origin and their respective roles in the thymic function. Furthermore, it remains to be determined whether different recirculating subsets could interact with distinct stromal cells and regulate the thymic selection, not only of Treg, but also of conventional and unconventional T cells. Moreover, recirculating Treg could participate to the homeostasis of the thymus similarly to their peripheral counterparts. Finally, future investigations aimed at deciphering the developmental pathways of specific functional Treg subsets are expected to elaborate personalized Treg therapies in the context of auto-immunity, inflammation, and regenerative medicine.

## Author Contributions

JS and MI conceived and wrote the manuscript. AB critically read and discussed the content prior to submission. All authors contributed to the article and approved the submitted version.

## Conflict of Interest

The authors declare that the research was conducted in the absence of any commercial or financial relationships that could be construed as a potential conflict of interest.
